# Whole-Genome Sequence of Endophytic Bacteria Associated with Poison Ivy Vine (Toxicodendron radicans)

**DOI:** 10.1128/mra.01232-22

**Published:** 2023-03-15

**Authors:** Han Ming Gan, Trevor S. Penix, Peter C. Wengert, Narayan H. Wong, André O. Hudson, Girish Kumar, Michael A. Savka

**Affiliations:** a Department of Biological Sciences, Sunway University, Subang Jaya, Malaysia; b Patriot Biotech Sdn Bhd, Subang Jaya, Malaysia; c Thomas H. Gosnell School of Life Sciences, Rochester Institute of Technology, Rochester, New York, USA; Wellesley College

## Abstract

Here, we report the genome assemblies of 11 endophytic bacteria, isolated from poison ivy vine (Toxicodendron radicans). Five species belonging to the genus Pseudomonas, two species of *Curtobacterium*, one strain of Pantoea agglomerans, and one species from the *Bacillus*, *Cellulomonas*, and Enterobacter genera were isolated from the interior tissue of poison ivy.

## ANNOUNCEMENT

Toxicodendron radicans, the poison ivy vine (PIV), is a member of the Anacardiaceae family and is native to central and eastern North America. It causes urushiol-induced contact dermatitis in most people (1). Urushiol is an oleoresin within the sap of the vine (2). Besides causing an immune response that leads to dermatitis (3), microorganisms harbored by the vine may cause secondary bacterial infection in persons that come in contact with the PIV (4). Therefore, sequencing of bacteria associated with PIV is important in the identification of bacteria that can lead to secondary human infections.

Here, we present the genome sequences of 11 PIV endophytes isolated in June 2018 from internal stem tissue taken from a rural site in Wheatland (43.033506, −77.806655), New York. Stem tissue of four pooled PIV plants was collected in early May (early growing season). Vines were surface sterilized, and the internal stem tissue was prepared axenically, inoculated in tryptic soy broth (TS) medium, incubated at 28°C, and cultured for 3 days, followed by plating and incubation under the same conditions on TS agar media to isolate culturable bacterial endophytes. Eleven morphologically distinct colonies were subcultured on TS agar medium at 28°C for 48 h to purity, and genomic DNA (gDNA) isolation was performed using the E.Z.N.A. bacterial DNA kit (Omega Bio-Tek, Norcross, GA) on 25 mg of cell mass. Each gDNA sample was processed and barcoded with a unique Nextera dual-index combination using the Nextera XT library preparation kit (Illumina, San Diego, CA) according to the manufacturer’s instructions. The libraries were pooled in equimolar concentrations and sequenced on the Illumina MiSeq platform (2 × 300-bp paired-end read configuration). In total, 30.8 million paired-end reads were generated for the 11 samples.

**FIG 1:**
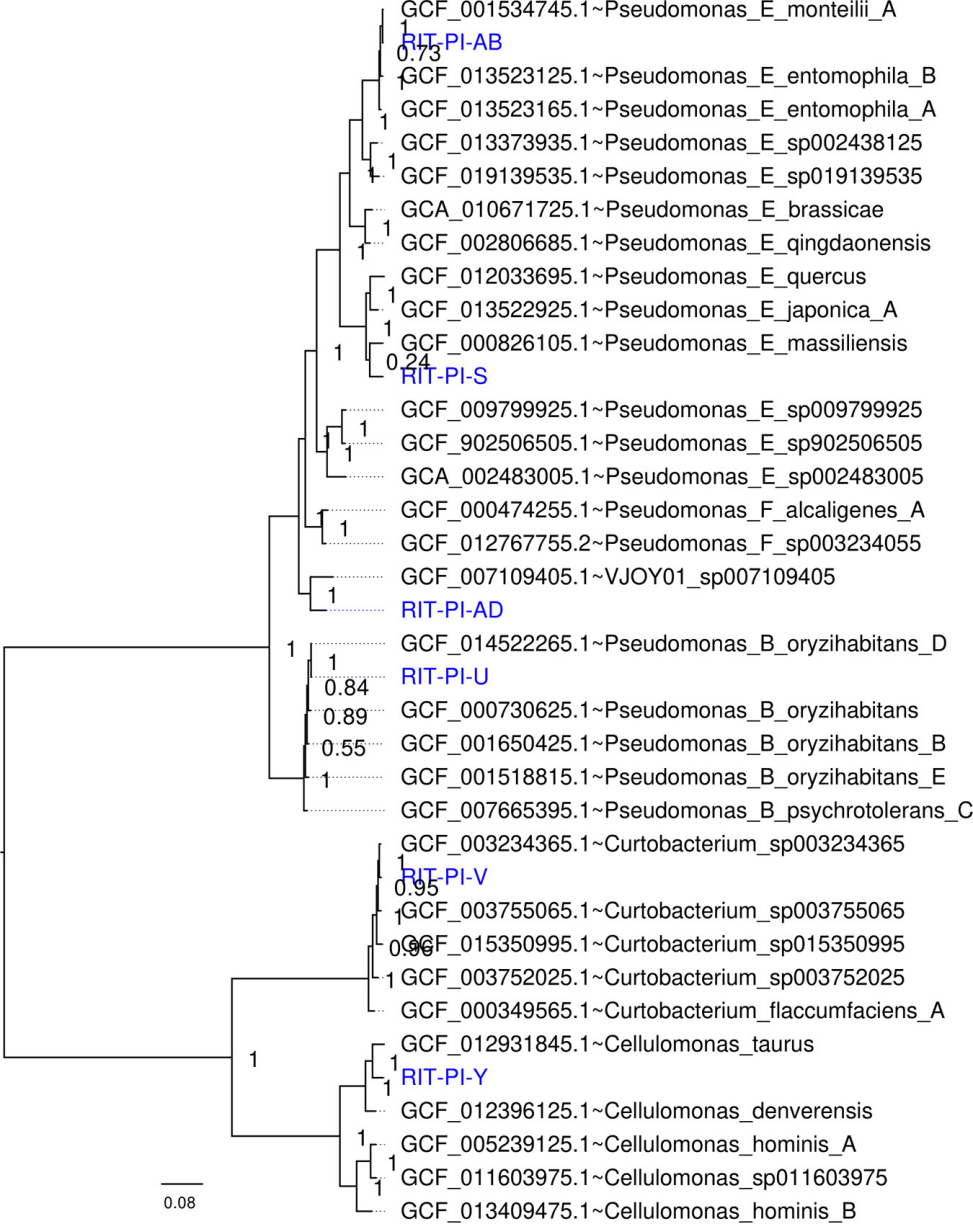
Phylogenomic tree of PIV strains unclassified at the species level. Maximum likelihood tree depicting the evolutionary relationships among sequenced poison ivy isolates without species classification (blue taxa) and their closely related strains in GTDB. The GTOTree pipeline (10) identified and aligned 74 single-copy bacterial genes from each genome assembly using hmmsearch version 3.3.2 (14) and MUSCLE version 3.8.1551 (15), respectively. The concatenated amino acid alignment was used to construct a maximum likelihood tree with FastTree version 2.1.10 (16) (default Whelan and Goldman [WAG] model for amino acid evolution). Branch lengths indicate the number of substitutions per site while node labels indicate Shimodaira-Hasegawa(SH)-like support values.

Default parameters were used for all software unless otherwise specified. Raw FASTQ reads for each library were adapter trimmed using Trimmomatic version 0.33 (5) and subsequently error corrected and *de novo* assembled into contigs with the SPAdes genome assembler (version 2.5.0) (6). To obtain quality statistics of the resulting assemblies, we used the quality assessment tool (QUAST; version 5.2.0) (7). FastANI version 1.33 (8) was used to calculate the pairwise average nucleotide identity of assembled genome against the representative genomes in the Genome Taxonomy Database (GTDB) r207 (9). The key annotation properties for the 11 genomes and taxonomic information are presented in Table 1. In addition, strains that were unclassified at the species level (<95% average nucleotide identity [ANI] to described species) were further analyzed using the GTOTree version 1.7.00 (10) (Fig. 1).

**TABLE 1:** Genome annotation information for the 11 PIV strains^*a*^

Strain	SRA accession	No. of reads (millions)	Total no. of bases (Mb)	Mean read length (bp)	Assembly accession	Assigned taxonomy	Best hit to GTDB (RefSeq accession)	%ANI	%cov	No. of contigs	Assembly length (Mb)	GC content (%)	*N*_50_ (kbp)	Coverage (×)	No. of CDS
RIT-PI-AB	SRS14903532	1.28	712	279	JANZKS01	Pseudomonas monteilii	Pseudomonas *E monteilii A* (GCF001534745.1)	98.5	90.9	82	4.7	64.3	150	152	4,159
RIT-PI-AC	SRS14903525	1.57	794	254	JANZKT01	Enterobacter huaxiensis	Enterobacter *huaxiensis* (GCF003594935.1)	98.9	93.2	58	5.12	55.56	533	155	4,870
RIT-PI-AD	SRS14903523	1.13	634	280	JANZKU01	Pseudomonas sp.	Pseudomonas *sp007109405* (GCF007109405.1)	85.4	75.7	84	4.81	66.2	160	132	4,369
RIT-PI-S	SRS14903521	1.22	665	273	JANZKJ01	Pseudomonas sp.	Pseudomonas *E japonica A* (GCF013522925.1)	82.2	59.7	50	4.89	63.02	261	136	4,377
RIT-PI-T	SRS14903522	1.37	703	256	JANZKK01	Pantoea agglomerans	Pantoea agglomerans (GCF001598475.1)	98.5	94.1	46	4.96	54.98	361	142	4,606
RIT-PI-U	SRS14903524	1.26	672	266	JANZKL01	Pseudomonas oryzihabitans	Pseudomonas *B oryzihabitans D* (GCF014522265.1)	97.9	91.9	38	5.02	65.83	258	134	4,616
RIT-PI-V	SRS14903526	1.22	655	269	JANZKM01	*Curtobacterium* sp.	*Curtobacterium sp003234365* (GCF003234365.1)	96.4	82.5	54	3.65	70.85	100	180	3,485
RIT-PI-W	SRS14903527	1.28	651	254	JANZKN01	Bacillus subtilis	Bacillus subtilis (GCF000009045.1)	98.7	91.7	32	4.31	43.32	571	151	4,423
RIT-PI-X	SRS14903528	1.32	702	265	JANZKO01	Pseudomonas atacamensis	Pseudomonas *E atacamensis* (GCF004801935.1)	96.9	88.2	74	5.91	60.12	240	119	5,344
RIT-PI-Y	SRS14903529	1.26	694	276	JANZKP01	*Cellulomonas* sp.	*Cellulomonas taurus* (GCF012931845.1)	87.6	75.1	94	3.52	72.69	57	197	3,302
RIT-PI-Z	SRS14903530	1.3	697	267	JANZKQ01	Pantoea agglomerans	Pantoea agglomerans (GCF001598475.1)	98.6	94.6	31	4.8	55.22	390	145	4,399

a % ANI, % average nucleotide identity; % cov, % genome query coverage; CDS, protein-coding sequences.

The four Pseudomonas sp. strains (RIT-PI-AB, RIT-PI-S, RIT-PI-AD, and RIT-PI-U) were clustered into distinct clades indicating high intergenomic diversity. Strain RIT-PI-U formed a monophyletic cluster with various genomospecies of Pseudomonas oryzihabitans, consistent with its high genome-wide ANI (gANI) to *P. oryzihabitans* (Fig. 1, Table 1). The analysis also supported the genus assignment of RIT-PI-V and RIT-PI-Y based on their placement within clades containing members of the same genus (Fig. 1). Members of the species Pantoea agglomerans and *P. oryzihabitans* are known to cause human opportunistic infections (11–13).

### Data availability.

The BioProject identifier (ID) registered for this project is PRJNA875050. NCBI accessions to the raw demultiplexed reads and genome assemblies were presented in Table 1.
